# Occupational Future Time Perspective: Psychometric Properties of a Spanish Scale

**DOI:** 10.3389/fpsyg.2018.02237

**Published:** 2018-11-20

**Authors:** Gabriela Topa, Hannes Zacher

**Affiliations:** ^1^Department of Social and Organizational Psychology, Universidad Nacional de Educación a Distancia, Madrid, Spain; ^2^Institute of Psychology, Leipzig University, Leipzig, Germany

**Keywords:** occupational future time perspective, motivation to continue working, retirement intentions, late career, retirement

## Abstract

Occupational future time perspective (OFTP) describes people’s perceptions of their future in the work context. In this study, we examined the psychometric properties of a Spanish OFTP scale (OFTP-SP). Data came from two samples of workers in Spain aged between 21 and 62 years (Study 1; *N* = 496) and between 40 and 70 years (Study 2; *N* = 386). In Study 1, we conducted descriptive analyses for the items and exploratory factor analysis. In Study 2, we conducted confirmatory factor analyses (CFA). Convergent validity of the OFTP-SP was examined based on relationships with employees’ motivation to continue working and retirement intentions. Results showed that reliability estimates were adequate, and hypotheses regarding the convergent validity for the three factors of the OFTP-SP (i.e., perceived remaining time, focus on opportunities, focus on limitations) were supported. The OFTP-SP is a psychometrically sound measure that can be used in future research on work and aging.

## Introduction

In the context of demographic, social, and economic changes, many governments are lifting retirement ages or making retirement entry more flexible (Organisation for Economic Co-operation and Development [OECD], 2018). The extension of working lives offers an opportunity for late career development to older workers. At the same time, it has been proposed as a way to retain the knowledge and experience of older workers, and as a promising tool to deal with international trends toward longevity (Sweet et al., 2017). Proactive career management requires individuals to adopt a long-term perspective that allows them to plan and prepare for their occupational future (Mooney et al., 2017). Over the last decade, occupational future time perspective (hereafter, OFTP) has emerged as a relevant construct to predict important work outcomes, such as job satisfaction, work engagement, and performance (Zacher and Frese, 2009, 2011; Zacher, 2013). Despite the growing body of empirical research, a systematic review (Henry et al., 2017), and a recent meta-analysis on OFTP (Rudolph et al., 2018), two caveats should be highlighted. First, most empirical studies were conducted with English-, German-, or Dutch-speaking samples, whereas no research has focused on workers in Spain, nor has a Spanish OFTP scale been developed. Second, the negative association between age and OFTP has been firmly established (Henry et al., 2017; Rudolph et al., 2018), but associations of OFTP with both for motivation to continue working and retirement intentions have so far been neglected.

Socioemotional selectivity theory states that people’s perception of time, including length of future time, as well as opportunities and limitations in the future, changes as a function of aging (Lang and Carstensen, 2002; Carstensen, 2006). Therefore, individual differences in future time perspective in the occupational context, or OFTP, could serve as a predictor of people’s tendency to pursue longer working engagement, expressed by intentions to delay retirement or motivation to continue working. While some antecedents of motivation to continue working seem to be rather stable, such as personality traits or sociodemographic characteristics, OFTP is more malleable and thus could be explored as an avenue to intervene with the goal of expanding occupational horizons of older workers.

Hence, this paper attempts to achieve two goals: on the one hand, we aim to adapt the OFTP scale for Spanish speaking employees and to test its psychometric properties with workers of different ages in Spain. On the other hand, we explore the convergent validity of OFTP for late career development indicators, that is, motivation to continue working and retirement intentions.

In Spain, the reforms of the Public Pensions System during 2011 and 2013 postponed the legal retirement ages and adopted a pension revaluation index to improve the sustainability of the system. As these changes led to a progressive reduction of the average pension value, estimated about 30% less between 2010 and 2050 (Sánchez-Martín, 2014), research on the factors associated to working longer intentions and behaviors seems be crucial both for individuals and society.

The present study will contribute to our understanding of OFTP among Spanish-speaking workers, as it will make available to researchers an instrument validated in Spanish. At the same time, it will provide evidence of the relationship between OFTP and late career workers’ intentions and developmental motivations. Policy makers, companies interested in retaining senior talent, and counseling psychologists need consistent empirical evidence to help employees extend their occupational life in meaningful ways, if they wish to do so (Pitt-Catsouphes et al., 2017).

### Late Career Development and OFTP

Late career development is the set of “work-related choices and reactions of people from 50 to 70 years of age and the economic, social, and organizational factors that influence them” (Greller and Simpson, 1999, p. 310). Late career development is subject to multiple influences that operate at the economic, legal, organizational, family, and personal levels (Voelpel et al., 2012). At the personal level, late career development seems directly related to the person’s age. However, chronological age shows weak and inconsistent relations with retirement intention and motivations to continue working (Ekerdt et al., 2000). In contrast, the perception of having many years to live, or *future time perspective* (Carstensen, 2006), has been shown to be linked to greater motivation and more desirable work outcomes, regardless of chronological age (Akkermans et al., 2016).

From this perspective, OFTP (Zacher and Frese, 2009) has been proposed as a key variable to understand the career development of older workers. It is defined as a person’s perception of his or her future time *in the work context*. Thus, OFTP is a cognitive-motivational characteristic that varies over time and with age (Carstensen, 2006; Cate and John, 2007) and includes three dimensions: perceived remaining time, focus on opportunities, and focus on limitations (Zacher, 2013). The first dimension, perceived remaining time, involves how much time individuals believe is left in the work or employment setting before leaving their working lives. Focus on opportunities includes the perceptions of goals, opportunities, and possibilities still available to the person in the work setting, whereas the focus on limitations focuses on the limitations and constraints in one’s work-related future.

Zacher and Frese (2009) adapted six (out of 10) context-free items from Carstensen and Lang (1996) general FTP scale to the work context and provided evidence for the reliability and validity of an OFTP measure with two distinct dimensions: perceived remaining time and focus on opportunities. Specifically, these two OFTP dimensions had internal consistency estimates of greater than 0.80 and differential relationships with demographic, personality, and work characteristics. In a subsequent study, Zacher (2013) adapted all 10 items from Carstensen and Lang (1996) to the occupational context and showed that OFTP can be measured reliably as a higher-order construct with three distinct dimensions: perceived remaining time, focus on opportunities, and focus on limitations. Zacher (2013) provided support for the validity of these three dimensions by showing that they are differentially associated with age, proactive personality, and job search intensity among older job seekers. Further evidence for the three-dimensional structure of the construct comes from a study by Rohr et al. (2017), who also showed that general FTP measured with Carstensen and Lang (1996) scale consists of the three dimensions identified by Zacher (2013). English, German, and Dutch versions of the OFTP scale have been used in empirical research (see Henry et al., 2017). We are not aware of systematic validation efforts of the OFTP scale in other countries. Importantly, we do not assume that OFTP is an emic construct that applies only in one cultural group. Instead, we assume that OFTP is an etic construct that can be applied to working people in all cultures that allow older workers to eventually retire from their work.

### Motivation to Continue Working and Retirement Intentions

Among the positive indicators of late career development is the motivation to extend working life beyond the traditional retirement age. This may entail the intention of engaging in bridge employment or in other forms of transitioning into full retirement, such as senior entrepreneurship. In contrast, retirement intentions involve that employees plan to retire sooner than later from their career job. In a qualitative review of the literature on OFTP, Henry et al. (2017) identified 16 published empirical studies that had examined relations between OFTP and these indicators of late career development. For instance, global OFTP was related to lower intention to retire (*r* = -0.33), as were focus on opportunities (*r* = -0.25), and perceived remaining time (*r* = -0.35; Bal et al., 2015). The dimension focus on opportunities shows the strongest association with the motivation to continue working beyond retirement age (*r* = 0.18; Zacher and Yang, 2016). On another hand, the dimension perceived remaining time is positively associated with learning motivation (*r* = 0.32), whereas focus on opportunities is related to related to achievement motivation (*r* = 0.32; Kooij and Zacher, 2016). The subsequent meta-analysis of Rudolph et al. (2018) extended the number of primary studies and confirmed the consistency of the relations between OFTP and late career development indicators.

Even though the number of empirical studies is still limited, the evidence is promising, as OFTP seems to interact with other variables, influencing employee outcomes that can promote late career development, such as the motivation to continue working, the decrease of intentions to retire, and the intensity of older people’s job search (Zacher, 2013).

In sum, we expect that the Spanish OFTP scale consists of three dimensions, perceived remaining time, focus on opportunities, and focus on limitations. Moreover, we expect these dimensions of OFTP to be positive related to motivation to continue working, and negatively related to retirement intentions.

## Materials and Methods

### Ethics Statement

The Institutional Ethics Committee of the first author’s university (National Distance Education University, UNED) approved this research on February 19th, 2018.

### Participants

We recruited two samples for this study. The first one (Sample 1) comprised workers in Spain aged between 21 and 62 years (*N* = 496), and the second one (Sample 2) included workers in Spain over 40 and under 65 years (*N* = 386). In Sample 1, mean age was 42.16 (*SD* = 9.8), 58.5% were female, and mean job tenure was 13.2 years (*SD* = 9.9). In Sample 2, mean age was 49.98 (*SD* = 6.7), 51.6% were male, and mean job tenure was 17.3 years (*SD* = 11.2). Regarding educational levels, in Sample 1, 7.1% had primary education, 7.9% secondary education, 24.2% vocational training, and 60.9% a university degree. In Sample 2, 19.4% had primary education, 11.4% secondary education, 14.8% vocational training, and 54.4% a university degree. Concerning occupational fields, in Sample 1, 1% worked in banking and finances, 18.1% in industry, 0.4% in telecommunications, and 80.4% in the services sector. In Sample 2, 9.6% worked in banking and finances, 24.9% in industry, 8.3% in telecommunications, 35.5% in education and health, and 80.4% in the services sector. Related to occupational categories, in Sample 1, 5.6% were managers, 12.1% were middle managers, and 73.3% were professional workers. In Sample 2, 13.5% were managers, 23.5% middle managers, and 49% were professional workers.

### Measures

All the scales were rated on 5-point Likert scales ranging from 1 (*strongly disagree*) to 5 (*strongly agree*).

#### Occupational Future Time Perspective

The OFTP scale (Zacher and Frese, 2009; Zacher, 2013), translated to Spanish, was used in this study. The instrument included ten items that were based on the future time perspective scale developed by Carstensen and Lang (1996) and Lang and Carstensen (2002) and adapted to the employment context by Zacher and Frese (2009). Previous exploratory factorial analyses (Zacher, 2013) confirmed a three-factor solution (i.e., perceived remaining time, focus on opportunities, and focus on limitations). Examples of items are: “My occupational future is filled with possibilities” and “There is plenty of time left in my occupational life to make new plans.”

#### Motivations to Continue Working

This variable was assessed with three items from Armstrong-Stassen (2008) scale, which were translated to Spanish by a bilingual translator to reflect a desire to continue working after retirement age. The questionnaire has also been adapted by the research group from the original version, which focused on working for the same organization, in order to express intention to continue working in a more general sense. The items are “Barring unforeseen circumstances, I would remain working as long as possible,” “I expect to continue working as long as possible after my retirement age,” and “If I were completely free to choose, I would prefer to continue working after my retirement age.” The reliability in our second study was 0.95.

#### Retirement Intentions

Based Adams and Beehr (1998), this variable was assessed with four items, which were translated from English to Spanish by a bilingual translator. The first item focuses on pension acceptance, the second, third and fourth reflect plans, desires, and expectations of retiring soon. Specifically, the items are: “I would like to retire in the near future,” “I expect to begin collecting a pension in the near future,” “I plan to retire in the near future,” and “I expect to retire in the near future.” The reliability in our second study was 0.88.

Sociodemographic data: Age (in years), gender (1 = Male, 2 = Female), education (1 = primary, 2 = secondary, 3 = vocational training, 4 = university degree), professional category (1 = managers, 2 = middle managers, 3 = professional workers, 4 = unqualified workers), and job tenure (in years) were measured with single items.

### Procedure

The translation and adaptation procedure were performed according to the guidelines suggested by Beaton et al. (2000), and included the following steps: translation and adaptation, synthesis, back-translation, and committee review. During all the phases, the bilingual translators were informed that the goal was to obtain a cross-cultural adaptation of the questionnaire. The first forward translation of the OFTP into Spanish was independently performed by two bilingual professionals; and the two translations were compared and discussed both by the two professionals and the research team to achieve the final version of the scale. Using the first version of the Spanish OFTP, two independent back-translations into English were performed. Subsequently, the research team analyzed the discrepancies with the original OFTP and resolved them by consensus, thereby creating the final OFTP-SP version of the scale.

Data collection for the first study (Sample 1) was carried out by means of questionnaires distributed in different organizations by collaborators of the research team in exchange for practical academic credits, and after receiving precise instructions to homogenize the administration of the tests. Participants were informed of the goals of the study, and the anonymity of the data collected. After they had expressed their consent, they completed the workbook containing the diverse scales of the study. For the second study (Sample 2), the research group e-mailed twelve firms to propose a broad study on human resources management. Ten firms responded and were then contacted by researchers to explain the criteria for the inclusion of participants (current employees aged above 40 years). Only eight organizations finally took part in the study. From the organizations, 489 current employees aged 40 or over received the questionnaire, a letter explaining the purpose of the study and the data collection procedure, as well as an envelope to return the survey. We collected 391 completed questionnaires (response rate of 80.5%).

### Data Analysis

First, to prevent the problem of missing data, collaborators of the research team have been instructed about carefully collecting data and monitoring missing data during the course of the study. Hence, sample 1 has no missing data. Regarding sample 2, as the proportion of missing data were less than 5%, Little’s MCAR (missing completely at random) test has been applied. The analysis showed that missing data values were completely at random (χ^2^= 18.822; *df* = 16, *p* = 0.278). In the following analyses, missing data were replaced by EM (expectation maximization) imputation in SPSS. Second, we conducted descriptive analysis for the items considering their central tendency and deviation measures (mean and standard deviation) and also their variability, by means of the Skewness and Kurtosis values. Skewness is considered an assessment of symmetry, while kurtosis is described as measure of whether the data are distributed relative to a normal curve. Third, we carried out an exploratory factor analysis (EFA) with Sample 1, using SPSS 25; and confirmatory factor analysis (CFA) with Sample 2, using Amos 25. We tested a model with three intercorrelated factors and then a model with one factor, which showed poorer fit to the data. Then, we tested the convergent validity by examining relationships of OFTP with retirement intentions and general motivation to continue by fitting a Structural Equation model using AMOS 25.

## Results

### Descriptive Statistics

Table 1 shows the descriptive statistics for the 10 items of the OFTP-SP. Means, standard deviations, skewness, and kurtosis for each item are included. The values for asymmetry and kurtosis between -2 and +2 are considered acceptable in order to prove normal univariate distribution (George and Mallery, 2010). All values of the OFTP-SP items were below 1 for skewness and for kurtosis.

**Table 1:** Descriptive statistics for OFTP-SP items Sample 1 (*N* = 496).

Items (English version)	Items (Spanish version)	*M*	*SD*	Skewness	Kurtosis
(1) Many opportunities await me in my occupational future.	(1) Me esperan muchas oportunidades en mi futuro laboral.	2.98	0.999	-0.110	-0.513
(2) I expect to set many new goals in my occupational future.	(2) Espero fijarme muchos nuevos objetivos en mi futuro laboral.	3.43	0.974	-0.412	-0.247
(3) My occupational future is full of possibilities.	(3) Mi futuro laboral está lleno de posibilidades.	3.13	0.980	-0.179	-0.302
(4) I could do whatever I like in my occupational future.	(4) Podría hacer lo que quisiera en mi futuro laboral.	2.83	0.919	0.002	-0.136
(5) I only have limited possibilities in my occupational future.	(5) Tengo solo posibilidades limitadas en mi futuro laboral.	2.97	0.924	-0.041	-0.495
(6) I have lots of time to make new plans for my occupational life.	(6) Queda mucho tiempo en mi vida laboral para hacer nuevos planes.	3.29	0.912	-0.324	-0.138
(7) Most of my occupational life lies before me.	(7) Tengo por delante de mí la mayor parte de mi vida laboral.	3.19	0.996	-0.157	-0.510
(8) My occupational future seems infinite to me.	(8) Mi futuro laboral me parece infinito.	2.65	0.892	0.243	-0.199
(9) I have the feeling that my occupational time is running out.	(9) Tengo la sensación de que mi tiempo laboral se me está acabando.	2.36	0.951	0.592	0.146
(10) As I get older, I have the feeling that my occupational time is limited.	(10) A medida que me hago mayor, tengo la sensación de que mi tiempo laboral es limitado.	2.71	1.010	0.172	-0.596


### Exploratory and Confirmatory Factor Analyses

First, we analyzed the psychometric properties of the OFTP-SP scale in Sample 1. In several studies, total-item correlation serves as a criterion for initial assessment and refinement. Following the criteria of Loiacono et al. (2002), items found to have low correlations (less than 0.40) with the total score were removed. All items showed an item-total correlation ranging from 0.74 to 0.50, except for Item 5, which showed a value of 0.34 and, according to Nunnally and Bernstein (1994) criteria, was excluded (see also Zacher, 2013). EFA was conducted with principle axis factoring and direct oblique (Oblimin) rotation. Based on previous research that examined the three dimensions of FTP and OFTP (i.e., perceived remaining time, focus on opportunities, focus on limitations; see Zacher, 2013; Rohr et al., 2017), we expected the dimensions of OFTP to be moderately to highly correlated and, therefore, used oblique rotation. The Kaiser-Meyer-Olkin (KMO) index (0.84) and Bartlett’s sphericity test [χ^2^(45, *N* = 496) = 2518.7, *p* < 0.000] indicated the appropriateness of EFA. The KMO criterion supported the three-dimensional solution, with eigenvalues >1. The four items loading on Factor 1 (*focus on opportunities*) reflected “individuals’ perceptions of their remaining goals, opportunities, and possibilities in the employment context” (Zacher, 2013, p. 1142). The three items included in Factor 2 (*perceived remaining time*) referred to the perceptions of “how much time an individual believes he or she has left in the occupational and employment context before exiting the labor market” (Zacher, 2013, p. 1142). Finally, the two items loading on Factor 3 (*focus on limitations*) included “individuals’ perceptions of the constraints, limitations, and restrictions in the employment context” (Zacher, 2013, p. 1142). All items loaded above 0.40 on their factor and below 0.30 on the other factors, as shown in Table 2.

**Table 2:** OFTP-SP: factor loadings for items Sample 1 (*N* = 496).

Items (English version)	Items (Spanish version)	Factor		
		1	2	3
(1) Many opportunities await me in my occupational future.	(1) Me esperan muchas oportunidades en mi futuro laboral.	0.791	0.116	0.057
(2) I expect to set many new goals in my occupational future.	(2) Espero fijarme muchos nuevos objetivos en mi futuro laboral.	0.612	0.209	-0.015
(3) My occupational future is full of possibilities.	(3) Mi futuro laboral está lleno de posibilidades.	0.939	0.020	0.031
(4) I could do whatever I like in my occupational future.	(4) Podría hacer lo que quisiera en mi futuro laboral.	0.737	0.073	0.046
(5) I only have limited possibilities in my occupational future.	(5) Tengo solo posibilidades limitadas en mi futuro laboral.	-0.347	0.137	0.250
(6) I have lots of time to make new plans for my occupational life.	(6) Queda mucho tiempo en mi vida laboral para hacer nuevos planes.	0.123	0.705	-0.060
(7) Most of my occupational life lies before me.	(7) Tengo por delante de mí la mayor parte de mi vida laboral.	0.008	0.850	-0.145
(8) My occupational future seems infinite to me.	(8) Mi futuro laboral me parece infinito.	0.204	0.447	-0.049
(9) I have the feeling that my occupational time is running out.	(9) Tengo la sensación de que mi tiempo laboral se me está acabando.	0.067	-0.245	0.734
(10) As I get older, I have the feeling that my occupational time is limited.	(10) A medida que me hago mayor, tengo la sensación de que mi tiempo laboral es limitado.	-0.005	-0.040	0.814
Explained variance of factor (total 60.8)		44.1%	6.3%	10.3%
Cronbach’s alpha		0.88	0.81	0.79


Item 5, which showed very low factor loadings on the three factors, has been excluded in the following calculations.

**FIGURE 1:**
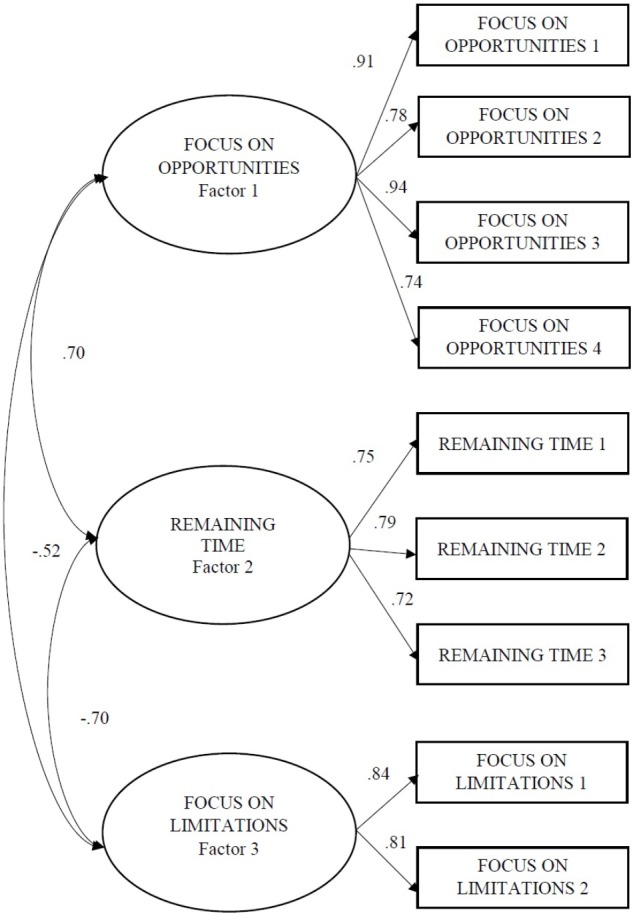
Confirmatory factorial analysis of OFTP-SP (Sample 2, *N* = 386).

To test the fit of the three-factor solution obtained by EFA, we conducted CFA with Sample 2, using the maximum likelihood procedure. We used the goodness-of-fit index (GFI), the adjusted goodness-of-fit index (AGFI), the comparative fit index (CFI), the incremental fit index (IFI), and the Tucker-Lewis index (TLI), which should all exceed 0.90. Additionally, and the root mean square residual (RMR) should be below 0.08. Our results met all the requirements to conclude that the three-dimensional theoretical model had a good fit: χ^2^(24, *N* = 386) = 91.3232, GFI = 0.9510, AGFI = 0.9082, CFI = 0.9674, IFI = 0.9675, TLI = 0.9510, RMR = 0.0563, RMSEA = 0.0854. Figure 1 shows the standardized estimates for the model.

Considering competitive hypotheses is an important phase of gathering evidence to support the internal structure of a scale. For this purpose, we compared the fit of the three-factor model [χ^2^(24, *N* = 386) = 91.3232, GFI = 0.9510, AGFI = 0.9082, CFI = 0.9674, IFI = 0.9675, TLI = 0.9510, RMR = 0.0563; RMSEA = 0.0854] with that of a single-factor model [χ^2^(27, *N* = 386) = 446.9273, GFI = 0.7606, CFI = 0.7964, NFI = 0.7870, IFI = 0.7973, TLI = 0.7285, RMSEA = 0.2010] for the same data. Differences between models were significant (Δχ^2^ = 355.6, *p* < 0.001); therefore, we rejected the most parsimonious model and supported the three-factor solution.

### Reliability

In Sample 1, Cronbach’s alpha reliability coefficients of the three subscales were adequate (ranging from 0.80 to 0.88, see Table 2), despite their reduced number of items. These values were confirmed in Sample 2 (ranging from 0.90 to 0.81).

### Interrelations of the Scales

The intercorrelations between the OFTP-SP subscales were moderate in size. Intercorrelations between the subscales in Sample 1 were: focus on opportunities-perceived remaining time (*r* = 0.58), focus on opportunities-focus on limitations (*r* = -0.37), and perceived remaining time-focus on limitations (*r* = -0.49). In Sample 2, intercorrelations were: focus on opportunities-perceived remaining time (*r* = 0.63), focus on opportunities-focus on limitations (*r* = -0.47), and perceived remaining time-focus on limitations (*r* = -0.56).

We further tested the scale’s validity with CFA (Sample 2) following Fornell and Larcker (1981) recommendation. The analysis of average variance extracted (AVE) reflects the total quantity of variance of the indicators tapped by the latent construct, and recommended values should be higher than 0.50. In this study, the AVE for Factor 1 (focus on opportunities) was 0.77; for Factor 2 (perceived remaining time), it was 0.71; and for Factor 3 (focus on limitations), it was 0.84.

To address the limitations of Cronbach’s alpha coefficient, CFA factor loadings can be used to provide a more accurate estimation of reliability through composite reliability (CR), developed by Werts et al. (1974). Scores should be higher than 0.70. In this study, the CR value was 0.93, 0.88, and 0.91 for Factors 1, 2, and 3, respectively.

To assess discriminant validity between the constructs, the dominant approaches are Fornell and Larcker (1981) criterion and the examination of cross-loadings. But recently, some doubts have emerged about these approaches, and Henseler et al. (2015) recommended more rigorous methods, such as the Heterotrait-Monotrait (HTMT) matrix of correlations.

Following the Fornell-Larcker procedures, the square root of the AVE should be higher than the correlation between constructs. Our results showed that the square root of the AVE was 0.88, 0.84, and 0.92 for Factors 1, 2, and 3, respectively. In view of these data, the constructs assessed in the model have discriminant validity, considering that the square root of the AVE values largely exceeds the intercorrelation among constructs. Moreover, in the present study, applying the threshold criterion (Gold et al., 2001), which suggests that all the values of the HTMT matrix should be lower than 0.90, it is concluded that the constructs assessed in the model have discriminant validity, as Table 3 shows.

**Table 3:** Heterotrait-Monotrait ratios and CI bias corrected (2.5 and 97.5%).

	HTMT ratio	2.5% CI	97.5% CI
(1) Focus on opportunities → Focus on limitations	0.54	0.44	0.649
(2) Remaining time → Focus on limitations	0.70	0.613	0.784
(3) Remaining time → Focus on opportunities	0.75	0.686	0.809


Additionally, if we apply the most restrictive criterion of Kline (2011), which states that all the values included in the confidence interval should be lower than 0.85, all the constructs assessed in the model showed adequate discriminant validity. Lastly, we explored the nomological network of the OFTP-SP, examining the correlation matrix and conducting structural equation modeling. We found that the three factors of the OFTP-SP were significantly associated with the motivation to continue working and retirement intentions in the sample of workers over 40 years old (see Table 4).

**Table 4:** Pearson correlation matrix [above the diagonal, Sample 1 (*N* = 496), below the diagonal, Sample 2 (*N* = 386)].

	*1*	*2*	*3*	*4*	*5*
(1) Focus on opportunities	*0.90*	0.58^∗∗^	-0.37^∗∗^		
(2) Perceived remaining time	0.64^∗∗^	*0.80*	-0.49^∗∗^		
(3) Focus on limitations	-0.47^∗∗^	-0.56^∗∗^	*0.81*		
(4) Motivation to continue working	0.32^∗∗^	0.22^∗∗^	-0.14^∗∗^	*0.95*	
(5) Retirement intentions	-0.31^∗∗^	-0.33^∗∗^	0.38^∗∗^	-0.54^∗∗^	*0.88*


*^∗∗^*p* < 0.01; values in italics in the diagonal are Cronbach’s alphas.*

**FIGURE 2:**
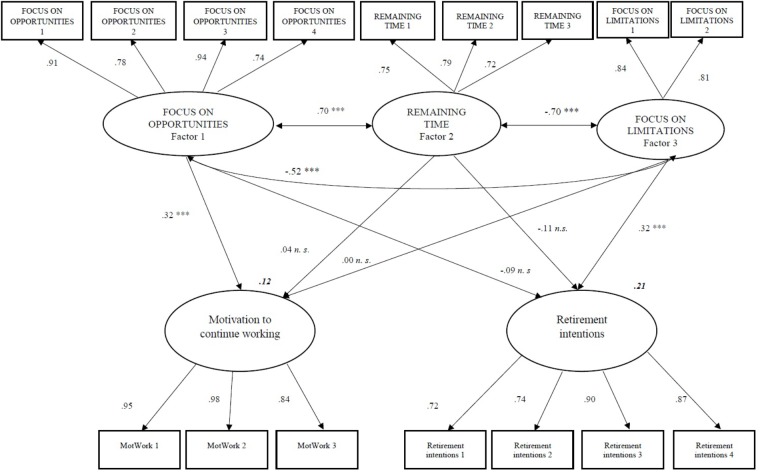
Standardized estimates for the SEM model (Sample 2, *N* = 386). ^∗∗∗^*p* < 0.001, *n. s*.: non-significant.

Next, we tested the convergent validity of the three OFTP-SP factors for motivation to continue working and retirement intentions, using the maximum likelihood procedure. The model presented a good fit: χ^2^(95, *N* = 386) = 379.3899, GFI = 0.9002, CFI = 0.9365, NFI = 0.9175, IFI = 0.9369, TLI = 0.9198, RMSEA = 0.0882. The critical ratios associated with some paths were not statistically significant (focus on limitations → motivation to continue working; perceived remaining time → motivation to continue working; focus on opportunities → retirement intentions). Model fit indicators, such as GFI, CFI, the normed fit index (NFI), IFI, and TLI should all be close to 0.90. Additionally, and the root mean square error of approximation (RMSEA) should be below 0.0882. Our results met all the requirements to conclude that the fit was adequate. Figure 2 shows the standardized estimates for the model.

## Discussion

There is a growing consensus about the role of future time perspective for people’s attitudes and behaviors, both in the work setting and in life in general (Kooij et al., 2018). Reviews of the literature on OFTP (Henry et al., 2017; Rudolph et al., 2018) confirm the convergent validity of the OFTP for motivation to continue working, retirement intentions, as well as task and contextual performance. In fact, the studies show that OFTP has predictive validity for attitudes and performance even after controlling for the influence of other constructs related to adaptation to aging, such as selection, optimization, and compensation strategies (Rudolph et al., 2018). Despite this, empirical studies have not analyzed these relations among workers in Spain. Specifically, to our knowledge, there is no study that has adapted the OFTP scale to Spanish language or analyzed its psychometric properties with workers in Spain. As the literature shows that OFTP is a relevant predictor associated with planning for and the decision to retire or to continue working (Topa et al., 2018), this analysis seems relevant.

In comparison with the original version in English, the version OFTP-SP presents adequate psychometric properties. The findings show that it is a valid and reliable tool to assess older people’s cognitive and affective expressions about their perceived remaining time in relation to their work. In addition, the present set of data provides evidence of the stability of its factor structure in different samples. This Spanish version would be useful for other Spanish-speaking populations, such as North, Central, and South America, even though cross-cultural research should be conducted in order to test the stability of its psychometric properties across countries.

The findings of the structural equation analysis reveal that the motivation to continue working beyond retirement is positively related to focus on opportunities, whereas the other two dimensions were not significantly related to this attitude. However, perceived remaining time and focus on limitations were associated with retirement intentions. However, we acknowledge that OFTP only accounts for a relatively small proportion of the variance in the two outcomes, as these attitudes and motivations are influenced by a multiplicity of antecedents at the societal, professional, organizational, and family level.

Concerning the size and representativeness of the samples in this study, the limitations of these data are obvious, especially regarding the sampling procedure used. Moreover, all the data proceed from self-reports, which can include a source of uncontrolled error from the common variance. However, as OFTP-SP is focused on subjective perceptions of occupational opportunities, limitations, and remaining time at work, deviations from external criteria would not necessarily indicate that it is invalid. In summary, we conclude that the available instrument could be used to expand research on OFTP and late career development among Spanish-speaking populations, and empirically support further theoretical development. In this regard, future analyses could expand empirical research to establish practical implications for subgroups of workers who show lower levels of OFTP. Subsequently, practical interventions aimed at improving future time attitudes among older workers could be developed (Hajek et al., 2018), for instance, as a way to reduce time to return to work among employees suffering from work-related stress (Björk et al., 2018).

Even though the number of empirical studies is still limited, the evidence is promising, as OFTP appears to interact with other variables, influencing behaviors that can promote late career development, such as older people’s job search intensity (Zacher, 2013). Finally, the possible cultural variations of OFTP should also be explored because temporal orientation is a dimension that characterizes and differentiates cultures, as noted by (Laureiro-Martinez et al., 2017).

## Ethics statement

This study has been approved by the Institutional Ethics Committee of the National Distance Education University (UNED). Written informed consent has been obtained from all participants.

## Author Contributions

GT and HZ designed the research. GT collected and analyzed the data. GT and HZ wrote and revised the manuscript.

## Conflict of Interest Statement

The authors declare that the research was conducted in the absence of any commercial or financial relationships that could be construed as a potential conflict of interest.
